# Two Novel Disease-Causing Mutations in the LDLR of Familial Hypercholesterolemia

**DOI:** 10.3389/fgene.2021.762587

**Published:** 2021-12-14

**Authors:** Haochang Hu, Tian Shu, Jun Ma, Ruoyu Chen, Jian Wang, Shuangshuang Wang, Shaoyi Lin, Xiaomin Chen

**Affiliations:** ^1^ Department of Cardiology, Ningbo First Hospital, Ningbo, China; ^2^ Department of Medical Ultrasonics, First Affiliated Hospital of Jinan University, Guangzhou, China

**Keywords:** familial hypercholesterolemia, low-density lipoprotein cholesterol, low-density lipoprotein receptor, disease-causing mutations, function

## Abstract

As an autosomal dominant disorder, familial hypercholesterolemia (FH) is mainly caused by pathogenic mutations in lipid metabolism-related genes. The aim of this study is to investigate the genetic mutations in FH patients and verify their pathogenicity. First of all, a pedigree investigation was conducted in one family diagnosed with FH using the Dutch Lipid Clinic Network criteria. The high-throughput sequencing was performed on three family members to explore genetic mutations. The effects of low-density lipoprotein receptor (*LDLR*) variants on their expression levels and activity were further validated by silico analysis and functional studies. The results revealed that LDLC levels of the proband and his daughter were abnormally elevated. The whole-exome sequencing and Sanger sequencing were used to confirm that there were two *LDLR* missense mutations (*LDLR* c.226 G > C, c.1003 G > T) in this family. Bioinformatic analysis (Mutationtaster) indicated that these two mutations might be disease-causing variants. *In vitro* experiments suggested that *LDLR* c.226 G > C and c.1003 G > T could attenuate the uptake of Dil-LDL by LDLR. In conclusion, the *LDLR* c.226 G > C and c.1003 G > T variants might be pathogenic for FH by causing uptake dysfunction of the LDLR.

## Introduction

Familial hypercholesterolemia (FH) is a common autosomal genetic disorder mainly caused by pathogenic mutations in genes encoding low-density lipoprotein receptor (*LDLR*), apolipoprotein B (*ApoB*) and proprotein convertase subtilisin kexin 9 (*PCSK9*) (Benn et al., 2016). A meta-analysis of 11 million subjects illustrated that the prevalence of FH in the general population is 0.32%, while the prevalence of FH in ischemic heart disease and premature ischemic heart disease patients is 3.2 and 6.7%, respectively (Beheshti et al., 2020). Due to lifelong exposure to extremely high levels of low-density lipoprotein cholesterol (LDLC), FH is characterized by xanthomas, corneal arcus, and early-onset cardiovascular disease (Peterson et al., 2021). European and American guidelines suggest that FH patients should be identified as early as possible so that LDLC lowering treatment can be started early in life in order to improve the patient’s prognosis (Piepoli et al., 2016; Grundy et al., 2019).

As a membrane protein on the surface of liver cells, LDLR is the key point of LDLC metabolism. LDLR could combine with LDLC and transport it to the lysosome for metabolism. The LDLR subsequently returns to the surface of liver cells for recycling (van de Sluis et al., 2017; Chemello et al., 2021). Therefore, the pathogenic variants in *LDLR* could directly lead to protein dysfunction and LDLC metabolic disorders. Previous studies suggested that the disease-causing variants in *LDLR* might account for about 90% of FH (Gidding et al., 2015; Iacocca and Hegele, 2017). Reeskamp *et al.* performed targeted next-generation sequencing on 1,528 patients with LDLC greater than 5 mmol/L. The results illustrated that 227 cases (14.9%) were heterozygous carriers of pathogenic variants in *LDLR* (80.2%), *APOB* (14.5%), and *PCSK9* (5.3%) (Reeskamp et al., 2021b). Furthermore, a retrospective study pointed out that LDLC gradually increased from patients with no pathogenic variants to patients with a defective variant, patients with a null variant, and patients with two variants (Di Taranto et al., 2021). Therefore, it is imperative to perform genetic diagnoses of FH patients to distinguish between different genetic states or variant types.

According to the UCL *LDLR* gene variant database, 3779 *LDLR* mutations have been reported so far; of which 77% are substitutions, 16% are deletions, and 5% are duplicates (Leigh et al., 2017). Regrettably, the pathogenicity of several *LDLR* variants has not been authenticated. It is crucial to research the functional verification of mutations to better clarify the molecular mechanism of FH.

In this study, a FH family was included, and high-throughput sequencing was used to explore pathogenic mutations. The effects of *LDLR* variants on the expression level and function of LDLR were verified in cellular experiments.

## Materials and Methods

### Patients

The study enrolled one FH family consisting of three members in Ningbo First Hospital. The Dutch Lipid Clinic Network criteria (DLCN) was used to diagnose FH patients (Wang et al., 2019). A detailed information collection form was designed based on the characteristics of FH patients. The content mainly included the patient’s general information, family history, personal history, past history, treatments, the results of the auxiliary examination, etc.

### Whole-Exome Sequencing and in Silico Analysis

Whole blood samples of the three participants were collected in EDTA tubes (Gongdong Company, China) and stored in a refrigerator at -80 °C (ThermoFisher Scientific, United States). Omega Blood DNA Kit (D3392-02, Omega bio-tek, United States) was used to extract genomic DNA. High-throughput whole-exome sequencing for DNA samples was completed on the BGISEQ-500 platform (Huada Gene Technology Co. Ltd., China). First, the genomic DNA was randomly broken into fragments of about 200–300 bp, and a complete fragment library was established after PCR amplification. The quality of DNA was then inspected before sequencing, and the number of original bases (raw data) obtained by sequencing each sample should meet the standard. Clean data was subsequently obtained by removing low-quality reads from raw data. Finally, we compared the sequencing data with the human reference genome hg19 to obtain high-confidence mutations.

As one of the standard bioinformatic tools, Mutationtaster (http://www.mutationtaster.org) was applied to evaluate the disease-causing potential of DNA variants (Schwarz et al., 2014). The transcript ID of the LDLR was ENST00000558518 (NCBI Reference Sequence: NM_000,527.5) in the manuscript.

### Sanger Sequencing

The PCR amplification method was utilized to amplify the participants’ DNA. PCR primers were designed based on the exon fragment where the mutation was located. The sequences of primers were shown as follows: *LDLR* (exon 3): 5′-TGA​CAG​TTC​AAT​CCT​GTC​TCT​TCT​G (upstream), 5′-ATA​GCA​AAG​GCA​GGG​CCA​CAC​TTA​C (downstream); *LDLR* (exon 7): 5′- AGT​CTG​CAT​CCC​TGG​CCC​TGC​GCA​G (upstream), AGG​GCT​CAG​TCC​ACC​GGG​GAA​TCA​C (downstream) (Du and Huang, 2007). Sanger sequencing was conducted to verify the variants in each participant by the Biosystems® 3730 DNA analyzer. The sequencing results were analyzed by the Chromas software.

### Cell Culture and Plasmid Transfection

HEK293 cells were derived from the cell bank of the Shanghai Chinese Academy of Sciences and were cultured in a DMEM medium (Hyclone, United States) with 10% fetal bovine serum (ThermoFisher Scientific, United States). The DMEM used in the current study included 4.00 mmol/L l-glutamine, 4500 mg/L glucose, and sodium pyruvate. After the cells were grown to about 80–90% in a 37 °C incubator containing 5% CO_2_, they were subcultured at a ratio of 1:3.

For plasmid transfection, HEK293 cells in good growth condition were plated in a six-well plate. In this study, wild-type (WT) and mutant plasmids were constructed by chemical synthesis. Lipofectamine 2000 reagent (ThermoFisher Scientific, United States), Opti-MEM medium (Gibco, United States), and plasmid DNA were mixed and incubated at room temperature and then added to the cells. The cells were cultured in 5% CO_2_ at 37 °C for 6–8 h.

We divided the cells into the mutant group (HEK293 cells transfected with LDLR mutant plasmids), the WT group (HEK293 cells transfected with LDLR wild-type plasmids), and the NC group (HEK293 cells transfected with empty plasmids).

### Expression of LDLR Variants

The expression level of LDLR variants was detected by Western Blot 48 h after plasmid transfection as previously described (Hu et al., 2021). The samples were lysed in RIPA buffer (Solarbio, China) containing protease and phosphatase inhibitors. Protein levels were quantified by the BCA protein assay kit (Cwbio, China). The samples containing equal amounts of protein were separated by 5x SDS-PAGE gel and transferred onto the PVDF membrane. After blocking with 5% milk, the samples were incubated with the primary antibodies: LDLR [Mouse monoclonal to LDL Receptor (Abcam, United States)] and *β*-actin [β-actin Rabbit mAb (Abclonal, China)] overnight at 4 °C, and secondary antibodies [peroxidase-conjugated anti-mouse IgG (Jackson Immuno Research, United States) and peroxidase-conjugated anti-rabbit IgG (Jackson Immuno Research, United States)] for 1 h at room temperature. Lastly, the signals were analyzed using the ImageJ Software.

### Uptake of Dil-LDL

After treatment with a serum-free medium of 0.3% bovine albumin (Solarbio, China) for 12 h, the transfected cells were incubated with 5 μg/ml Dil-LDL (Thermo Scientific, United States) for 4 h. The labeled LDL was used to study LDL uptake through endocytosis and its trafficking throughout the cell, which can be detected via fluorescence microscopy. After washing the cells with PBS, they were fixed with 4% paraformaldehyde for 10–20 min and then stained with DAPI. The fluorescence intensity of the cells was observed using a confocal laser scanning microscope (LEICA TCS SP8, Germany). The ImageJ software was used to quantitatively analyze the fluorescence intensity.

### Statistical Analysis

All statistical analyses were performed by the SPSS 24.0 software (SPSS Inc., Chicago, IL, United States). GraphPad Prism 8 (GraphPad Software, La Jolla, CA) was used for plots. *p* < 0.05 was considered statistically significant.

## Results

### The Clinical Information of the Proband and Pedigree Investigation

A 52 year-old man was diagnosed with coronary heart disease at Ningbo First Hospital. His LDLC level was abnormally elevated at 5.62 mmol/L under the treatment of atorvastatin 20 mg. Corneal arcus and xanthomas on the skin of the elbow were observed since adolescence. The results of coronary angiography illustrated 95% localized stenosis of the proximal segment of the left anterior descending branch, 90% localized stenosis of the proximal segment of the diagonal branch, and subtotal occlusion of the proximal segment of the right coronary artery. Echocardiography displayed hypertrophy of the ventricular septum, aortic valve calcification, and mild mitral regurgitation. Carotid ultrasound showed multiple plaque formation in bilateral carotid arteries.

We tracked three members in two generations of this family, including the proband’s wife and the proband’s daughter. As shown in Table 1, the blood lipid level of the proband’s wife (I-2) was normal. However, the proband’s daughter (II-1) had an increased level of LDLC (5.50 mmol/L) without corneal arcus or xanthomas. Before performing DNA analysis, the proband and his daughter were diagnosed as FH, according to the DLCN criteria.

**TABLE 1:** Clinical data of FH patients and family members.

Characteristics	I-1	I-2	II-1
Gender	Male	Female	Female
Age (year)	52	53	27
Triglycerides (mmol/L)	1.42	1.43	0.46
Total cholesterol (mmol/L)	7.64	3.78	7.36
High-density lipoprotein cholesterol (mmol/L)	0.91	1.49	1.95
Low-density lipoprotein cholesterol (mmol/L)	5.62*	2.25	5.50
ApoA1 (g/L)	0.97	1.68	1.50
ApoB (g/L)	1.64	0.64	1.42
Lipoprotein a (mg/dl)	36.90	42.40	15.50
Carotid plaque	Yes	No	No
Carotid stenosis	No	No	No
Aortic valve Calcification	Yes	No	No
Left ventricular ejection fraction (%)	68	70	70
Corneal arcus	Yes	No	No
Xanthoma	Yes	No	No
Coronary artery disease	Yes	No	No

The asterisk indicates that the patient is taking a lipid-lowering drug (atorvastatin 20 mg).

### Mutational Analysis, in Silico Analysis, and Sanger Sequencing

**FIGURE 1:**
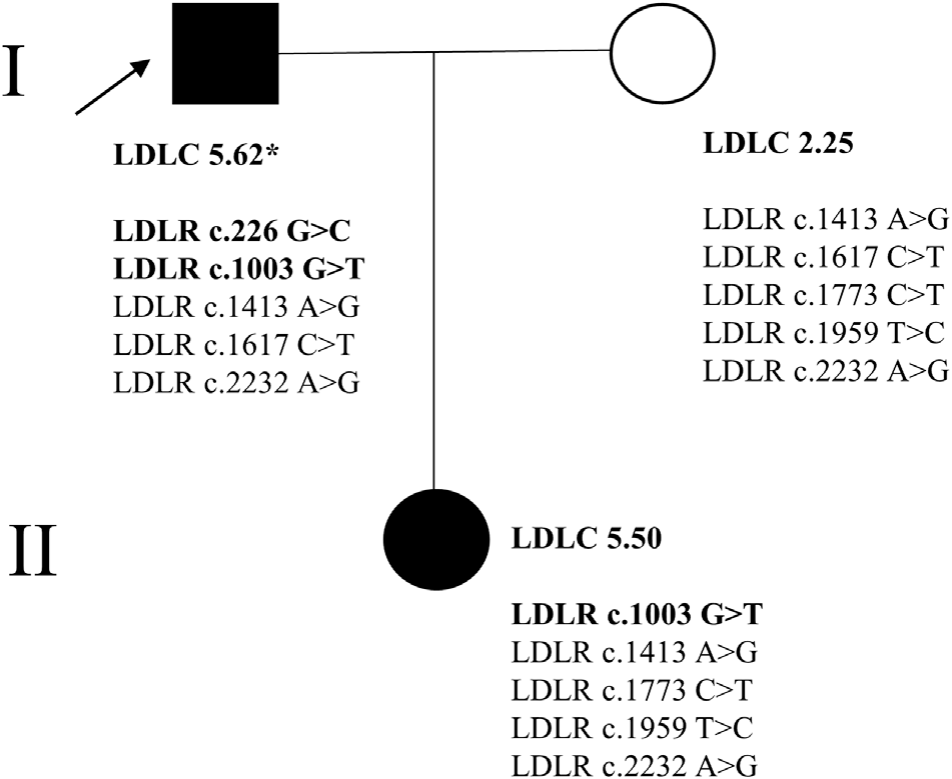
The family tree of the proband. * signified that the patient was taking the lipid-lowering drug (atorvastatin 20 mg); → represented the proband. In the current study, the background of the FH patients’ (I-1, II-1) picture was blackened. The disease-causing variants in LDLR were bolded.

High-throughput sequencing was performed on all members of this FH family, and the mutations of four FH-related genes (*LDLR, APOB, PCSK9, LDLRAP1*) were analyzed (Tada et al., 2019). There were five *LDLR* variants (*LDLR* c.226 G > C, c.1003 G > T, c.1413A > G, c.1617 C > T, and c.2232A > G) in the proband, five *LDLR* variants (*LDLR* c.1413A > G, c.1617 C > T, c.1773 C > T, c.1959T > C, and c.2232A > G) in the proband’s wife and five *LDLR* variants (*LDLR* c.1003 G > T, c.1413A > G, c.1773 C > T, c.1959T > C, and c.2232A > G) in the proband’s daughter (Figure 1). However, no suspicious disease-causing variant was identified in the other three genes.

The impact of *LDLR* variants on the receptor function was investigated by the bioinformatic tool Mutationtaster. The results revealed that *LDLR* c.1413A > G, c.1617 C > T, c.1773 C > T, c.1959T > C, and c.2232A > G were synonymous mutations, while *LDLR* c.226 G > C and c.1003 G > T were missense mutations. The amino acid sequences of these two sites were highly conserved in various species (Supplementary Figure S1).

In addition, both variants could cause changes in amino acid sequence. LDLR c.226 G > C (exon 3) caused the 76th amino acid to change from glycine to arginine, namely p. Gly76Arg. Meanwhile, LDLR c.1003 G > T (exon 7) caused the 335th amino acid to change from glycine to cysteine, namely p. Gly335Cys.

**FIGURE 2:**
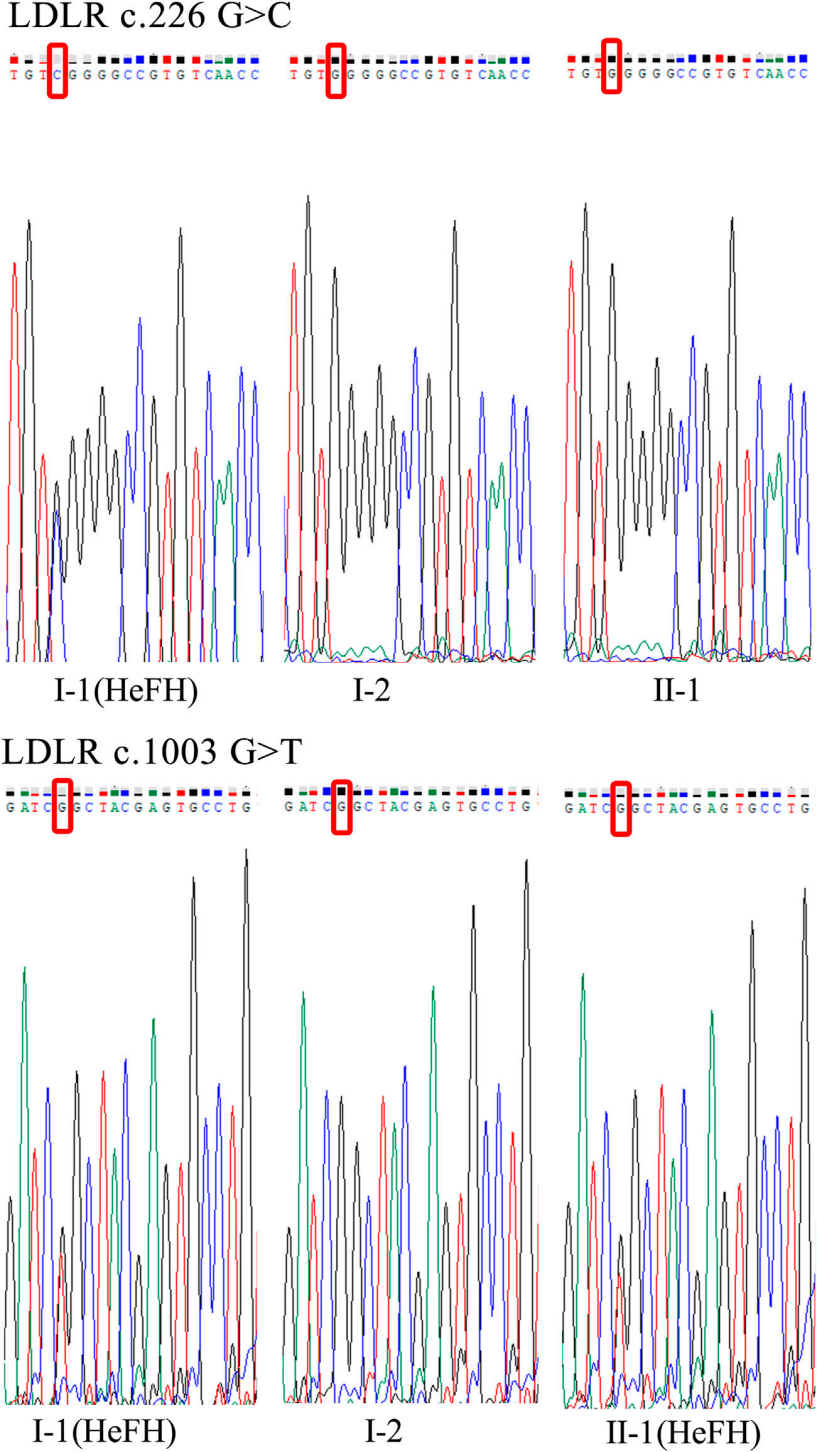
Target sequences on LDLR by Sanger sequencing. Two disease-causing LDLR variants (LDLR c.226 G > C, c.1003 G > T) were found in the proband (I-1). The LDLR c.1003 G > T variant was found in the proband’s daughter (II-1).

Subsequently, Sanger sequencing was performed to verify the existence of the two variants in the corresponding family members (Figure 2). The proband had two variants (*LDLR* c.226 G > C and c.1003 G > T), and his daughter had one variant (*LDLR* c.1003 G > T). Therefore, we speculate that the FH family in this study may be caused by pathogenic mutations in the *LDLR*.

### The Expression of LDLR Variants and Uptake of Dil-LDL

**FIGURE 3:**
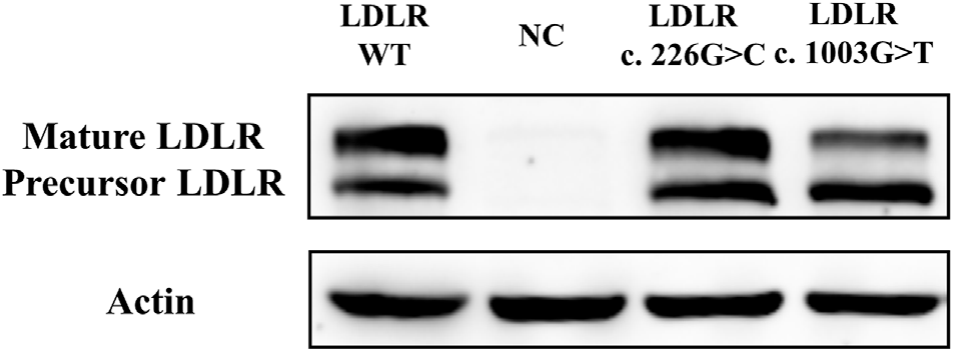
The effect of LDLR variants on LDLR expression.

The expression level of *LDLR* in the mutant group, WT group, and NC group was detected by Western Blot in HEK293 cells. The two bands detected in the gel map corresponded to the mature type and the precursor type LDLR, respectively. The results demonstrated that there was no significant difference between the mutant group (*LDLR* c.226 G > C) and the WT group (Figure 3). Compared with the WT group, less mature LDLR was detected in the *LDLR* c.1003 G > T group.

**FIGURE 4:**
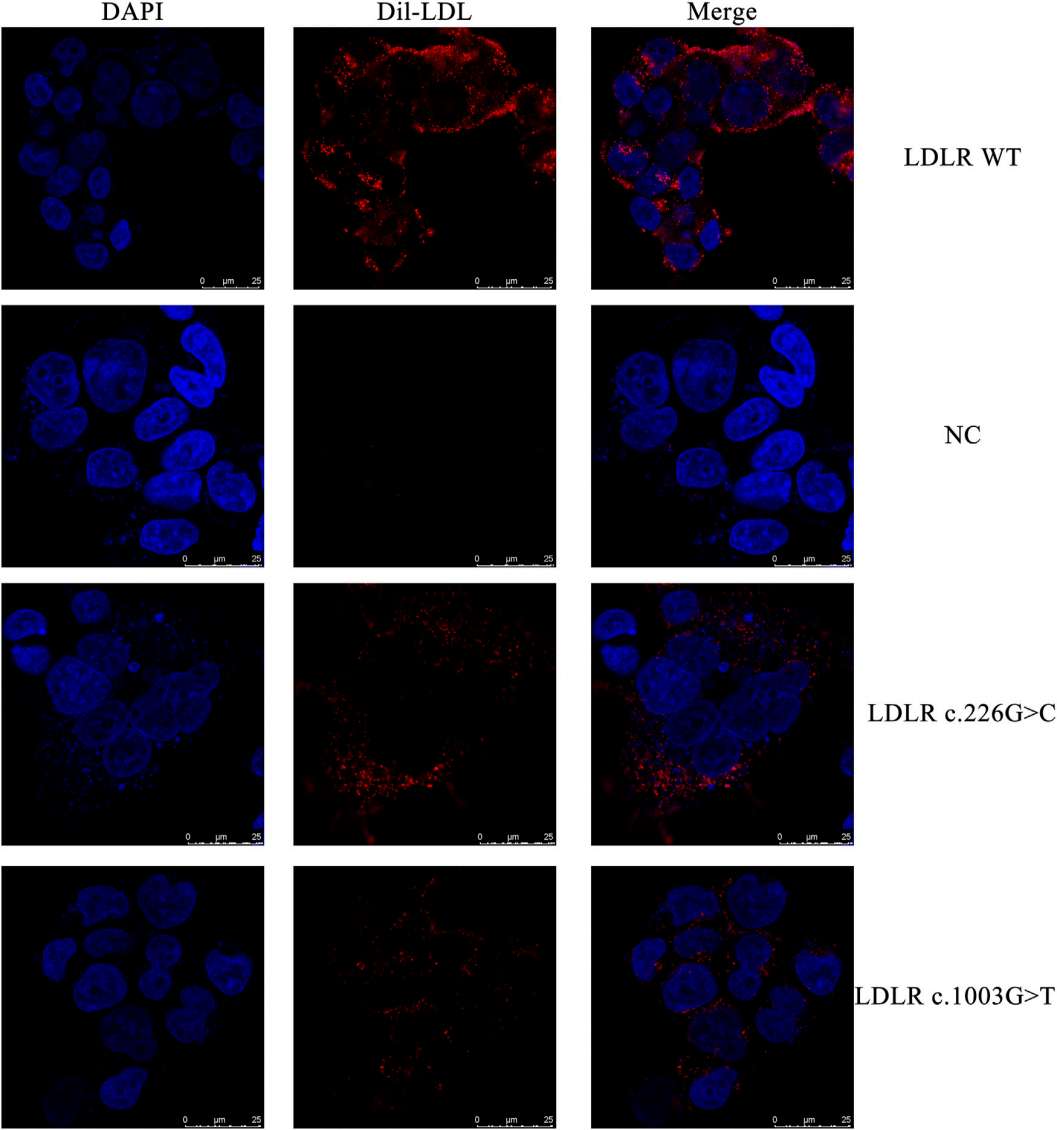
The representative confocal microscopy images. Blue fluorescence represented DAPI, while red fluorescence represented Dil-LDL.

**FIGURE 5:**
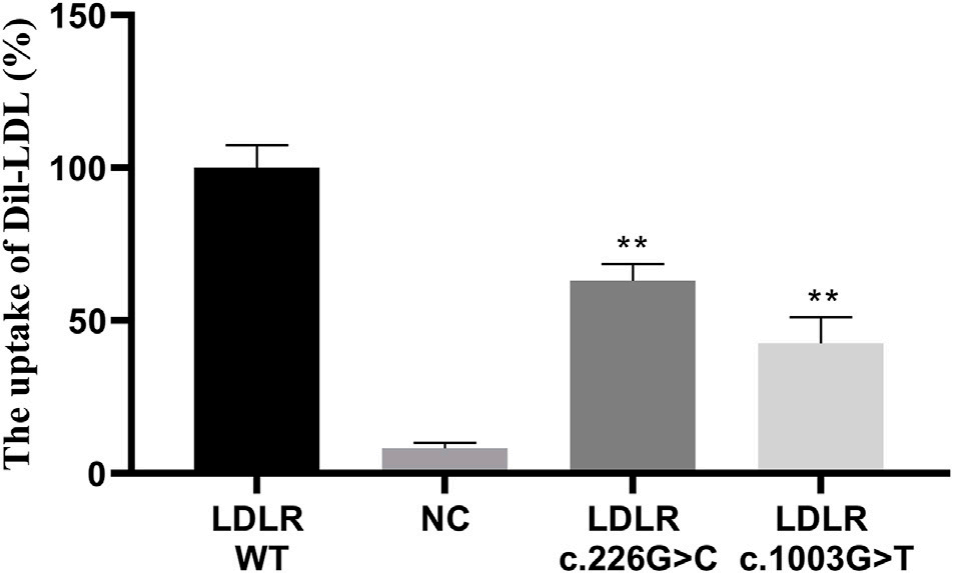
The LDL uptake in different LDLR groups. ** indicates statistical difference between mutant group and WT group (*p* < 0.01).

The HEK293 cells carrying the *LDLR* c.226 G > C and c.1003 G > T variants and the WT *LDLR* were incubated in Dil-LDL medium for 4 h. The ability of mutant LDLR (*LDLR* c.226 G > C, c.1003 G > T) to take up LDL was significantly lower than that of WT LDLR (*LDLR* WT: 100%, *LDLR* c.226 G > C: 63.04%, *LDLR* c.1003 G > T: 42.54%, *p* < 0.01, Figure 4, Figure 5).

### The Correlation Between the Phenotype and the LDLR Mutation

Judging from the FH patients’ clinical phenotype, the proband’s blood lipid level, corneal arcus, xanthoma, and atherosclerosis were severe. According to the sequencing results, the proband was a compound heterozygote (*LDLR* c.226 G > C and c.1003 G > T), and his daughter was a heterozygote (*LDLR* c.1003 G > T). This finding partly explained that the clinical phenotype of compound heterozygous patients was more severe than that of heterozygous.

## Discussion

As a typical disease of abnormal cholesterol metabolism, FH is a significant risk factor in the occurrence and development of cardiovascular disease (Hu et al., 2020). With the advancement of molecular technology, some FH patients have undergone genetic testing to elucidate the pathogenic mechanism. However, the current diagnostic rate of FH in most countries is extremely low, at < 1% even. Hence, it is crucial to carry out screening of high-risk populations in order to improve the diagnostic rate. Herein, a detailed pedigree investigation and the genogram of the proband and his family members were conducted. Two *LDLR* variants (*LDLR* c.226 G > C and c.1003 G > T) were discovered in this family by whole-exome sequencing. Functional prediction through the bioinformatic software showed that both variants might impact the expression or function of LDLR. The *in vitro* analysis confirmed that *LDLR* c.226 G > C and c.1003 G > T could diminish the ability of LDLR to uptake LDL.

By consulting the literature and the UCL database on LDLR mutations (http://www.lovd.nl/LDLR), we confirmed that both variants (*LDLR* c.226 G > C and c.1003 G > T) were not previously identified. The *LDLR* c.226 G > C variant (exon 3) is located in the coding region of the LDLR ligand-binding domain, which consists of 292 amino acids, including seven repeats (Strom et al., 2020). In the process of LDL metabolism, the variants including *LDLR* c.226 G > C in the LDLR ligand-binding domain might result in the LDLR being unable to reach the cell surface or in its inability to bind to LDL. This can eventually lead to hyperlipemia (Pamplona-Cunha et al., 2020). Our functional studies corroborated that *LDLR* c.226 G > C did not affect its expression. Instead, it caused the ability of LDLR to uptake LDL to decrease. In addition, another mutation, *LDLR* c.226 G > T (p. Gly76Trp), was also found at the exact location (Bourbon et al., 2008; Usifo et al., 2012). However, the expression and LDL internalization by *LDLR* c.226 G > T were similar to the WT, which was defined as likely benign (Benito-Vicente et al., 2015). It implies that different variants in the same position may function differently.

The *LDLR* c.1003 G > T variant (exon 7) is located in the homology domain of the EGF precursor, which consists of 406 amino acids, contains three EGF-like repeat units, and one *β*-propeller domain (Springer, 1998). It might result in the synthesized LDLR protein not being released from the endoplasmic reticulum to the cell surface, in that the receptors that reach the cell surface cannot bind to LDL, or in that the receptor cannot be recycled (Austin et al., 2004). Jeenduan *et al.* identified that LDLR p. D151Y and M391T located in the homology domain of EGF precursor significantly reduced the expression level of LDLR on the cell surface to 18 and 38%, respectively. Additionally, the amount of LDL uptake by LDLR was reduced to 15 and 71%, respectively (Jeenduang et al., 2010).

Our study showed that the other variant *LDLR* c.1003 G > T might affect the expression of the LDLR protein and impair its ability to uptake LDL (reduced to 42.54%). Previous studies have also reported the variant *LDLR* c.1003 G > A (p. Gly335Ser), and bioinformatics predicted that this variant was likely pathogenic (Wang et al., 2001; Laurie et al., 2004; Bertolini et al., 2013; Narang et al., 2020). Interestingly, two different variants in the translation initiation codon of LDLR (*LDLR* c.1A > T and c.1A > C) encoded the same amino acid (LDLR p.Met1Leu), but they cause different degrees of damage to its expression and activity (Graca et al., 2021). Therefore, functional experiments are paramount to clarify whether the mutation is pathogenic or not.

Ana Catarina Alves *et al.* found that a missense mutation (*LDLR* p. Asp601Val) might cause the loss of LDLR mature form and a complete impairment of its activity (Alves et al., 2021). The variant *LDLR* c. 2389 G > A might cause the erroneous cleavage of messenger RNA to retain the mutant LDLR in the Golgi apparatus (Shu et al., 2021). Existing evidence indicates that missense mutations in LDLR could affect its expression and cause dysfunction of LDLR protein through a variety of mechanisms. Regrettably, our research cannot identify the mechanism of the diminished ability of LDLR by two variants. Therefore, future research should focus on exploring the mechanism of FH caused by its variants.

Generally, the clinical phenotype of homozygous FH patients is more severe than that of heterozygous patients, whereby some patients might eventually suffer from cardiovascular events in adolescence or even childhood (Sanchez-Hernandez et al., 2016). Moreover, the double heterozygous carriers of autosomal dominant hypercholesterolemia gene mutations have an intermediate phenotype compared with heterozygous and homozygous/compound heterozygous carriers (Sjouke et al., 2016). In this study, the proband was a compound heterozygous FH, and his daughter was a heterozygous FH. Due to being exposed to high levels of LDLC at birth, FH patients often exhibit severe atherosclerosis with the time response and dose effect of LDLC (Ference et al., 2017). The LDLC level of the proband was exceptionally high, even after drug therapy, and there were severe manifestations such as corneal arcus, xanthomas, carotid artery stenosis, coronary artery stenosis, and aortic valve calcification. Comparatively, the daughter of the proband only had hypercholesterolemia without any other clinical phenotypes. The lipids*age was proposed as an indicator to predict the risk of arteriosclerotic cardiovascular disease (Ference et al., 2018). The influence of age should be considered on the FH patient’s phenotype. Furthermore, early introduction of lipid-lowering treatment and long-term medical management could reduce the occurrence of cardiovascular events for the proband’s daughter.

There are some limitations in the current study. On the one hand, only three members of one family participated in the study. On the other hand, we only detected the expression of LDLR in the whole cell lysate but did not detect the number of LDLR on the cell surface. In the future, attention should be paid to the effect of LDLR mutation on the remaining activity of LDLR to unravel the damage of LDLR mutation to its function. Finally, we mainly focused on the variants in the exon regions of LDLR in our study. It has been shown that the mutations in the intron regions of LDLR may affect the splicing of mRNA precursors and lead to the occurrence of FH (Reeskamp et al., 2021a). Further studies should be performed to explore the mechanism of LDLR intron region variation in the occurrence and development of FH.

In conclusion, two novel variants, *LDLR* c.226 G > C and c.1003 G > T, might be pathogenic for FH by causing LDLR uptake dysfunction.

## Data Availability

The datasets presented in this article are not readily available because according to the requirements of the Institutional Ethics Committee, the sharing of genomic data in the public domain is not allowed. Requests to access the datasets should be directed to the corresponding authors.
