# Supplementary figures and images for: Two Novel Disease-Causing Mutations in the LDLR of Familial Hypercholesterolemia
Source: Front Genet. 2021 Dec 14;12:762587. doi: 10.3389/fgene.2021.762587 (PMC8712701; doi:10.3389/fgene.2021.762587)

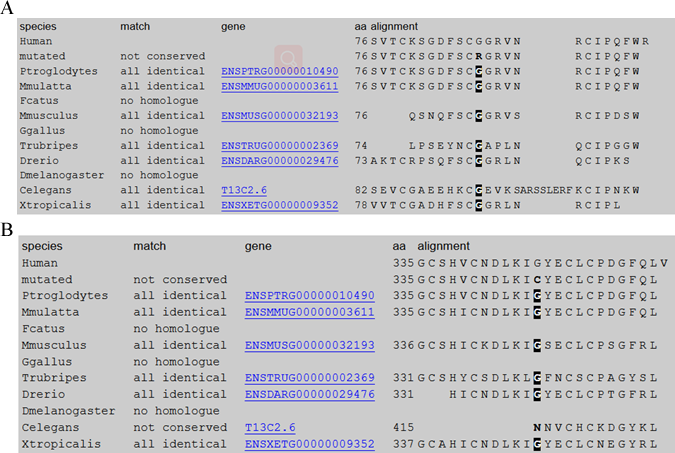

Supplement: Supplementary file 1 [file Image1.TIF]
